# The effect of the timing of antibiotics and surgical treatment on infection rates in open long-bone fractures: a 6-year prospective study after a change in policy

**DOI:** 10.1007/s11751-014-0208-9

**Published:** 2014-12-20

**Authors:** Andreas Leonidou, Zoltan Kiraly, Hristifor Gality, Shane Apperley, Sean Vanstone, David A. Woods

**Affiliations:** Department of Trauma and Orthopaedic Surgery, Great Western Hospitals NHS Foundation Trust, Marlborough Road, Swindon, SN3 6BB UK

**Keywords:** Open long-bone fracture, Time to theatre, Grade of surgeon, Infection rate

## Abstract

Our current protocol in treating open long-bone fractures includes early administration of intravenous antibiotics and surgery on a scheduled trauma list. This represents a change from a previous protocol where treatment as soon as possible after injury was carried out. This review reports the infection rates in the period 6 years after the start of this protocol. Two hundred and twenty open long-bone fractures were reviewed. Data collected included time of administration of antibiotics, time to theatre and seniority of surgeon involved. The patients were followed up until clinical or radiological union occurred or until a secondary procedure for non-union or infection was performed. Clinical, radiological and haematological signs of infection were documented. If present, infection was classified as deep or superficial. Surgical debridement was performed within 6 h of injury in 45 % of cases and after 6 h in 55 % of cases. Overall infection rates were 11 and 15.7 %, respectively (*p* = 0.49). The overall deep infection rate was 4.3 %. There was also no statistically significant difference in the subgroups of deep (*p* = 0.46) and superficial (*p* = 0.78) infection. Intravenous antibiotics were administered within 3 h of injury in 80 % of cases and after 3 h in 20 % of cases. The infection rates were 14 and 12.5 %, respectively (*p* = 1.0). There was no statistically significant difference in the subgroups of deep (*p* = 0.62) and superficial (*p* = 0.73) infection. Further statistical analysis did not reveal a significant difference in infection rates for any combination of timing of antibiotics and surgical debridement. Infection rates where the most senior surgeon present was a consultant were 9.5 % as opposed to 16 % with the consultant not present, but this trend was not statistically significant. These results suggest that the change in policy may have contributed to an improvement of the deep infection rate to 4.3 % from the previous figure of 8.5 % although this decrease is not statistically significant. Surgeons may have had concerns that delaying theatre may lead to an increased infection rate, but these results do not substantiate this concern.

## Introduction

Infection is a recognized complication of open fractures. Surgical debridement of open fractures has been conducted usually within 6 h of injury based on studies conducted by Leopold Freidrich that showed an increase in the growth of microbiological colonies after 6 h in a guinea pig model. In recent years, the validity of the 6-h rule has been questioned by studies showing no statistically significant increase in infection rates beyond 6 h providing that there is prompt administration of antibiotics and the wound is not overtly contaminated [1–3].

In 2009, the British Orthopaedic Association (BOA) in conjunction with the British Association of Plastic Reconstructive and Aesthetic Surgeons (BAPRAS) produced guidelines on the management of open long-bone fractures. Within the guidance, it is advocated that surgical debridement should be carried out by a senior orthopaedic surgeon within 24 h of injury unless contamination, compartment syndrome, vascular compromise or the open fracture being a part of multiple injuries necessitated earlier surgical intervention [4].

A change in the protocol to reflect the need for early administration of intravenous antibiotics (within 3 h of admission) as well as aiming to perform surgical debridement once the patient and surgical team were fully optimized, typically at the next consultant-led trauma list was instituted. This study reviews the infection rates since the protocol change in 2006 and asks whether early antibiotic administration and senior surgical care reduce infection in open long-bone fractures.

## Patients and method

All open long-bone fractures managed in our department from 1 January 2006 to 31 December 2011 were identified from records and studied. This is a prospective cohort study of a series of consecutive patients.

The initial management was aimed at providing:

1. A senior orthopaedic surgeon (registrar or above) involved in the care of the patient from the outset.
2. Wounds superficially cleaned, photographed and covered with an antiseptic-soaked dressing.
3. Fracture splintage.
4. Intravenous antibiotics within 3 h of the time of injury.
5. Anti-tetanus prophylaxis if indicated.
6. Urgent transfer to a level 1 trauma centre if a neurovascular injury or significant tissue loss requiring plastic surgical input was evident.

Patients not in the category above were added to the next available trauma theatre list for surgical wound debridement and initial or definitive fracture stabilization. This included patients with significant tissue loss (GA IIIB) that did not get transferred urgently to a level 1 trauma centre. In these cases, a wound debridement and application of external fixation were performed prior to the subsequent transfer to a tertiary centre for definitive management and appropriate coverage of soft tissue defects as required.

Specifically, there were no cases where the open fracture underwent internal fixation without prior soft tissue cover nor were any wounds left open for gradual spontaneous closure with granulation tissue.

There were 220 consecutive open long-bone fractures in 212 patients included in the study. Choice of intravenous antibiotic was as per trust protocol which had changed from Cefuroxime and Metronidazole to Augmentin as of September 2008. The fractures were stabilized using a variety of techniques depending on individual fracture patterns and the treating surgeon’s preference. The wounds were either closed primarily where appropriate or left open, and the patients were re-operated at 48 h for secondary debridement or closure.

Patients who died within 3 months of injury were excluded from the study. There was insufficient time from injury to death for the outcome of infection to be known. Patients who required transfer to a specialist unit for definitive treatment of the bony injury were excluded; this group included patients with more severe injuries (GA IIIB and IIIC) and patients who required treatment for both the bony and soft tissue injuries out with facilities of this hospital.

Follow-up was continued until clinical or radiological union, or a secondary procedure for non-union or infection was performed. The diagnosis of deep infection was made along the criteria set by Horan et al. [5]. More specifically, these were: purulent drainage from the deep incision; deep abscess formation; fascial dehiscence either by the infection or during reoperation; or deep infection in the presence of a metallic implant around bone [5]. Additionally, the diagnosis of deep or superficial infection was also based on radiological evidence and cultures obtained either at the time of a secondary procedure to treat infection or non-union, or from discharging wounds. All patients with a diagnosis of superficial wound infection were treated with oral antibiotics and the infection resolved.

The data on all open fractures were recorded in regular weekly audit meetings, attended by the duty trauma consultant, junior staff and members of the departmental clinical audit team. In each case, in addition to the age and gender of the patient, the following were recorded: (1) the site and severity of the fracture using the Gustilo Anderson classification (confirmed in theatre after debridement); (2) the time of admission to the emergency department or time from injury when possible; (3) the time of administration of antibiotics (> or <3 h); (4) the time to surgery (> or <6 h); (5) the grade of the most senior surgeon present in surgery; and (6) whether or not infection (deep vs. superficial) subsequently developed.

The collected data were statistically analysed using the two-tailed Fisher’s exact test with the significance level set at *p* = 0.05.

## Results

Two hundred and twenty fractures were identified. Two patients died within 3 months of the injury. A further 57 fractures were excluded from the study. Of these, 27 patients (47 %) were transferred to a tertiary hospital for plastic surgical care, 17 patients (30 %) were lost to follow-up (mostly due to living out of our area) and 13 patients (23 %) were excluded because of errors in data collection. As a result, the 161 remaining fractures in 75 female and 86 male patients were included in the study. The mean age of the patients was 45 years (range 8–92 years). According to the Gustilo Anderson Classification, 59 fractures were type I, 32 fractures type II, 40 fractures type IIIA and 30 fractures type IIIB (subsequently transferred).

The overall infection rate in the study group was 13.6 %, with 4.3 % deep infections and 9.3 % superficial. The number of infections in relation to timing of initial antibiotics and first surgery is shown in Table 1.

**Table 1:** Infection in open long-bone fractures in relation to timing of antibiotics and time to theatre from the injury

	Timing of antibiotics (h)		Timing of theatres (h)	
	<3	>3	<6	>6
No infection	111	28	64	75
	18 (14 %)	4 (12.5 %)	8 (11 %)	14 (15.7 %)
Infection				
Deep infection	5 (4 %)	2 (6.25 %)	2 (2.7 %)	5 (5.7 %)
Superficial infection	13 (10 %)	2 (6.25)	6 (8.3 %)	9 (10 %)

Surgical debridement was performed within 6 h of injury in 45 % of cases and after 6 h in 55 % of cases. Overall infection rates were 11 and 15.7 %, respectively, and was not statistically significant (*p* = 0.49). The deep infection rate in the patients operated within 6 h was 2.7 % and in the patients operated after 6 h 5.7 %. The superficial infection rate in the patients operated within 6 h was 8.3 % and in the patients operated after 6 h 10 %. There was no statistically significant difference in the subgroups of deep (*p* = 0.46) and superficial (*p* = 0.78) infection.

Intravenous antibiotics were administered as per protocol within 3 h of injury in 80 % of cases and after 3 h in 20 % of cases. Overall infection rates were 14 and 12.5 %, respectively, and was not statistically significant (*p* = 1). The deep infection rate in the patients with administered antibiotics within 3 h of injury was 4 % and in those after 3 h from injury 6.25 %. The superficial infection rate in the patients with administered antibiotics within 3 h of injury was 10 % and in those after 3 h from injury 6.25 %. There was no statistically significant difference in the subgroups of deep (*p* = 0.62) and superficial (*p* = 0.73) infection.

Further statistical analysis did not reveal any significant difference in infection rates for any combination of timing of antibiotics and time to first surgical debridement.

In 63 cases, the most senior surgeon present at the operation was a consultant and in the remaining 98 cases a non-consultant middle grade surgeon. Overall infection rates were 9.5 and 16 %, respectively, but this was not statistically significant (*p* = 0.24). The deep infection rate in the patients operated with a consultant present was 4.7 % and in the patients operated with a middle grade present 4 %. The superficial infection rate in the patients operated with a consultant present was 4.8 % and in the patients operated with a middle grade present 11 %. There was also no statistically significant difference in the subgroups of deep (*p* = 1) and superficial (*p* = 0.16) infection.

Our protocol for antibiotics in open long-bone fractures changed in September 2008 from intravenous Cefuroxime and Metronidazole to intravenous Co-amoxiclav. The overall infection rate before and after the implementation of the new policy was 18.2 and 7.3 %, respectively, and was not statistically significant (*p* = 0.06). There was also no statistically significant difference in the subgroups of deep (*p* = 0.7) and superficial (*p* = 0.09) infection. The detailed comparative data are presented in Table 2.

**Table 2:** Comparative data between the previous and current antibiotic policy

	Previous policy (cefuroxime + metronidazole)	Current policy (co-amoxiclav)
Number of cases	93	68
Deep infection	5 (5.3 %)	2 (3.1 %)
Superficial infection	12 (12.9 %)	3 (4.4 %)
Overall infection	17 (18.2 %)	5 (7.3 %)

The previously published data from our department before the change in the policy included 248 open long-bone fractures with a deep infection rate of 8.5 % [3]. From these patients, 62 % were operated within 6 h of injury and 38 % after 6 h [3]. The overall deep infection rate in our current study was 4.3 % which is lower but not statistically significant in comparison with our previous data (*p* = 0.16). A comparative presentation of the data before and after the change in policy is shown in Table 3.

**Table 3:** Comparative data before and after the change in the policy for managing open long-bone fractures in 2006

	Before the change	After the change
Number of cases	248	161
Time to theatre <6 h	154 (62 %)	94 (38 %)
Time to theatre >6 h	72 (45 %)	89 (55 %)
Deep infection cases	21 (8.5 %)	7 (4.3 %)

The isolated organisms in the cases of deep and superficial infection are shown in Table 4.

**Table 4:** Isolated organisms in cases of deep and superficial infection and number of cases

Deep infection	Superficial infection
Coagulase negative *Staphylococcus* [3]	*Staphylococcus aureus* [7]
*Staphylococcus aureus* [3]	*Enterococcus* [4]
Methilicinne resistant *Staphylococcus aureus* [2]	Mixed Coliform Bacilli [3]
Mixed Coliform Bacilli [3]	Diphtheroids [4]
*Enterococcus* [1]	Non-haemolytic *Streptococcus* [1]
Diphtheroids [1]	
*Escherichia coli* [1]	
Pseudomonas [1]	
*Enterobacter cloacae* [1]	
Mixed anaerobes [1]	

In order for the difference in deep infection rate to have been statistically significant and the study to have had a power of 0.80, our sample size should have been 834.

## Discussion

The results of the present study on the less severely injured open long-bone fractures demonstrate that following the change in our policy of early administration of antibiotics and operative management in the next organized trauma list, the deep infection rate has not increased; on the contrary, it has decreased although not to a statistically significant degree. Furthermore, timing of antibiotics and timing to theatre are not statistically correlated with the development of either deep or superficial infection. The grade of surgeon showed a trend of decreased infection rates with a consultant present; nevertheless, this was not statistically confirmed. It must be emphasized at the beginning of this discussion that we are not advocating delaying surgery in all cases. The most severe open long-bone fractures with associated neurovascular injury or tissue loss still remain a surgical emergency, and the current policy in the UK is to send all of these patients to a level 1 trauma centre.

The findings of this review are in agreement with our previously published results in 2007 where the conclusion of there being no significant correlation between the operation time (within or more than 6 h of injury) and the development of deep infection in patients with open long-bone fractures is held [3]. Several recent published studies convey the same conclusion. Crowley et al. [6] in their review paper commented on the lack of scientific evidence supportive of early surgery in open fractures, particularly with relation to the historical 6 h rule. Furthermore, Pollak et al. [2] on their prospective study of 315 patients with open fractures concluded that time from injury to operative debridement is not a significant independent predictor of the risk of infection. Additionally, Sungaran et al. [7] recommended delaying surgery on open tibia fractures until optimal operating environment can be provided. This was on the basis of their study in which 161 open tibia fractures were divided into three groups depending on the time to theatre. Interestingly, five infections occurred in the early operated (within 6 h) group, whereas only one infection occurred in the 6–12 h group and none in the 12–24 h group [7].

A recent meta-analysis by Schenker et al. [8] did not identify an association between delayed debridement of open long-bone fractures and the post-operative development of either deep or superficial infection. In addition to the aforementioned studies where time to theatre has been shown not to correlate with infective complications, several authors have studied the effect of after-hours surgery on patient’s morbidity and surgical outcome. More specifically, it has been suggested that after-hours surgery results in increased surgical complications, technical errors as well as increased reoperation rates [9–11]. Sleep deprivation and the subsequent decreased mental alertness and manual dexterity have been hypothesized to be responsible for these worse surgical outcomes during out-of-hours operating [10, 11].

The previously published data from our unit led to a change in our policy of managing open long-bone fractures. This change was in line with the most recent BOA/BAPRAS guidelines and reflected the need for early administration of intravenous antibiotics (within 3 h of admission) as well as performing surgical exploration and debridement only once the patient and surgical team were fully optimized, typically at the next consultant-led trauma list [4]. Comparing the results of the present study to our previously published data, it is of note that the deep infection rate has improved following the change in policy. More specifically, the deep infection rate in our previous study was found to be 8.5 % [3]. In the present study, the overall infection rate is 13.6 % with 4.3 % of infections being classified as deep. An improvement of 4.2 % in the deep infection rate occurred. It is of note that the change in the antibiotic policy resulted in a decrease in superficial infection rates but did not affect the deep infection rates as shown in Table 2. It is therefore suggested that the decrease in deep infection rates is not a result of the change in the administered antibiotic regime.

In cases where the most senior surgeon present was a consultant, the overall infection rates were 9.5 % as opposed to 16 % when the most senior surgeon was a non-consultant middle grade surgeon. Nevertheless, this trend was not statistically confirmed and was probably due to small number of patients in the sample. Harrison et al. [12] have recently investigated the effect of the grade of the surgeon in the development of deep infections following hip fracture surgery. The authors concluded that operations performed by a consultant or a hip fracture specialist had half the rate of the infection in comparison with non-consultant grades, and this was statistically confirmed [12]. Furthermore, Edwards et al. [13] have shown that more experienced surgeons have lower grade of infection in hip fracture surgery, but this was not statistically confirmed.

The strength of our study is that it is a retrospective review of a consecutive series on a large number of open long-bone fractures. The main limitation was that our study was underpowered as the desirable sample size to elicit a statistically significant difference at the deep infection rates was 834 fractures. We continue to collect data and may in future be able to statistically confirm any difference with larger numbers of patients. There are also many confounding factors which render the analysis of the reasons for any change in outcome difficult, such as age, patient co-morbidities, fracture configuration, mechanism of injury and surgical technique. Finally, 59 patients were lost to follow-up because of errors in data collection but most importantly because of transfer to a major trauma centre for further treatment. These cases transferred to a specialist unit were in the majority high-energy injuries with high Gustilo and Anderson grade.

## Conclusion

We have investigated the infection rate in open long-bone fractures not associated with neurovascular injury or severe tissue loss following a change in our management policy from treating these injuries as soon as possible to treat them on the next available trauma list when appropriately trained nursing and medical staffs are available. The overall infection rate was 13.6 %, with 4.3 % deep infections and 9.3 % superficial. The deep infection rate before the change in the policy was 8.5 %. The improvement in the deep infections rate was not affected by the change in the antibiotics policy. We have not yet analysed our data for other outcomes, such as ultimate healing of the fracture, number of subsequent operations or outcome for the limb, but we are able to suggest that delaying the surgery in order to maximize the whole operative team has not resulted in an increased deep infection rate and would support continued application of the BOA/BAPRAS guidelines.
